# Action of Monroe County Medical Society, Regarding Publication of State Society Transactions

**Published:** 1873-11

**Authors:** 


### Correspondence.

ROCHESTER, N. Y., October 8th, 1873.

DEAR DOCTOR:—Enclosed please find a copy of the resolutions adopted at the special meeting of the Monroe County Medical Society on the 6th inst. The action of the society was suggested by that of the Medical Society of New York, and these resolutions are similar to those adopted by that organization. You will find a brief notice of their meeting in the Oct. Number of the N. Y. Med. Record. I trust that the other County Societies may view the matter from the same point, and take such action as will enable the Publishing Committee to publish the transactions of the late meeting before another is due.

Very Sincerely, Yours, E. V. STODDARD.

*Whereas*, No provision for the publication of the transactions of the last meeting of our State Medical Society was made by the Legislature of the State of New York.

*Resolved*, That it is the sentiment of the members of the Monroe County Medical Society, that the committee on publication of the State Medical Society should adopt measures to secure the prompt publication of the transactions at the expense of the County Societies.

*Resolved,* That this Society will meet its proportionate amount of such expense.

*Resolved,* That a copy of these resolutions be forwarded to the President and to the Publishing Committee of the State Society.

Adopted, October 6th, 1873.

